# Contemporary cohort study in adult patients with infective endocarditis

**DOI:** 10.1016/j.bjid.2025.104521

**Published:** 2025-04-02

**Authors:** Mariana Giorgi Barroso de Carvalho, Thatyane Veloso de Paula Amaral de Almeida, Nicolas de Albuquerque Pereira Feijóo, Rafael Quaresma Garrido, Giovanna lanini Ferraiuoli Barbosa, Wilma Félix Golebiovski, Bruno Zappa, Clara Weksler, Marcelo Goulart Correia, Cristiane da Cruz Lamas

**Affiliations:** a Universidade do Grande Rio/Afya (UNIGRANRIO/Afya), Departamento de Medicina, Rio de Janeiro, RJ, Brazil; b Instituto Nacional de Cardiologia, Rio de Janeiro, RJ, Brazil; c Instituto Nacional de Infectologia Evandro Chagas (Fiocruz), Rio de Janeiro, RJ, Brazil

**Keywords:** Infective endocarditis, Epidemiology, Cohort study, Surgery, Mortality, Brazil

## Abstract

**Background:**

Infective Endocarditis (IE) is a serious disease, with high morbidity and mortality. Few case series come from middle- income countries. Our aim is to describe a case series of patients with IE treated at a reference center in Brazil and compare data to other countries.

**Methods:**

A retrospective analysis of a prospectively implemented endocarditis database was conducted, including adult patients with definite IE, January 2006‒June 2023. A literature search and summary were done. Statistical analysis was performed using Jamovi®, version 1.2.2.

**Results:**

There were 502 episodes of IE; mean age ± SD was 48.4±17.2 years. Community-acquired IE occurred in 64.7 %. Main predispositions were rheumatic valve disease (30.7 %), prostheses (31.5 %), and congenital heart disease (13.9 %). Transthoracic and transesophageal echocardiograms were performed in 85 % and 78 %, respectively. Left-sided IE predominated. Fever occurred in 90.6 %, new murmurs in 50.7 %, and embolism in 45 %. Blood cultures were negative in 33.1 %; frequent pathogens were oral streptococci (15.6 %), *Staphylococcus aureus* (10.0 %), and enterococci (12.8 %). Main complications were heart failure (58 %), renal failure (32.8 %), and splenic embolization (35.2 %). Surgery was performed in 83.6 %; overall in-hospital mortality was 25 %; surgical mortality was 21.3 %.

**Conclusions:**

Blood culture negative left-sided IE predominated. The mortality rate was high but within the range of reported series. Surgery was performed frequently and patients who were operated on had lower mortality.

## Highlights

• Infective endocarditis in adults often affected younger individuals.
• Rheumatic valve disease and prosthesis were common predispositions.
• Blood culture negative, oral streptococci and enterococcal endocarditis were frequent.
• Mortality was high.

## Introduction

Infective Endocarditis (IE) is an infection of the endocardium, the lining surface of the heart chambers, primarily affecting the cardiac valves (native or prosthetic), non-valvular endocardial surfaces, and intracardiac devices. It is a serious condition with high morbidity and mortality rates, leading to complications such as heart failure and embolism [1].

The epidemiology of IE varies depending on host factors and causative agents. In recent decades, despite improvements in diagnostic and therapeutic strategies, the incidence of IE has been increasing worldwide. This increase may be due to longer life expectancy, improved healthcare access, and increased use of cardiac devices and implants [1,2] Consequently, the epidemiology in high-income countries has significantly shifted, with an increase in older patients affected by IE, Prosthetic Valve Endocarditis (PVE), and device-related endocarditis within this population. However, studies in low- and middle-income countries still highlight Rheumatic Valve Disease (RVD) and uncorrected congenital heart diseases as common predisposing factors [3,4]

Therefore, the aim of our study was to analyze the epidemiological, clinical, microbiological, echocardiographic characteristics, as well as complications and outcomes of IE over the past 17-years at a tertiary hospital in Rio de Janeiro (Brazil), and to compare these findings with those of other series from low-, middle-, and high-income countries.

## Material and methods

### Study scenario

The study site, Instituto Nacional de Cardiologia, is a public tertiary care medical centre offering high-complexity cardiology care and cardiac surgery, including heart transplantation and, more recently, lung transplantation, in the city of Rio de Janeiro, Brazil. The institute has 165 beds, 45 of which are adult intensive care beds, and it is a teaching hospital, training cardiology and cardiac surgery residents. Annually, it performs approximately 1200 surgeries and conducts 50,000 medical outpatient consultations. The Department of Heart Valve Diseases provided an average of 5989 outpatient consultations annually between 2019 and 2023, with an average of 207 valve surgeries per year during this time span.

### Study population and study design

This was a prospective observational cohort study of consecutive adult patients with IE hospitalized at institution. The study included only adult patients meeting the definite criteria for IE according to the modified Duke criteria, [6] spanning from 1 January 2006 to 30 June 2023. Study variables were collected using standardized International Collaboration on Endocarditis case report forms as previously described [2] Data analysis was conducted *post hoc*.

### Variables analyzed

Data included patient demographics, epidemiological and clinical data, underlying cardiac conditions, comorbidities, and predisposing factors for IE. Clinical manifestations, complications, causative microorganisms, echocardiographic findings, surgeries, and outcomes were assessed. Laboratory parameters such as erythrocyte sedimentation rate, C-Reactive Protein (CRP) levels, rheumatoid factor, and urinalysis results were obtained.

### Literature review

A literature search was conducted using the virtual library databases PubMed and Virtual Health Library (Biblioteca Virtual em Saúde, BVS). The following descriptors were used: ‘endocarditis’, ‘epidemiology’, ‘cohort studies’, and their respective terms in Portuguese and Spanish. The inclusion criteria were epidemiological studies from the last 10-years (2014–2024), with a minimum sample size of 100 participants, most of whom fulfilled definite IE according to the modified Duke criteria. The exclusion criteria were multicentre studies, studies published more than 10-years ago, and studies with a sample size of less than 100 participants. These data were used to build a Supplementary Table used in the discussion. However, large multicenter series were also read and used in the discussion.

### Definitions

The definitions of IE were based on the modified Duke criteria, as well as guidelines from the European Society of Cardiology (ESC) in 2015 and 2023 for the management of IE [1,5,6]

Community-acquired and healthcare-associated IE were defined according to the ESC 2009 guidelines [7]. Individuals with healthcare-associated IE were categorized as those manifesting IE 48 h after hospital admission or those who acquired IE through an invasive procedure performed within the past 8-weeks before symptom development [1,6] Early PVE was defined as that occurring within 1-year of heart valve surgery. Late PVE was defined as occurring 12 or more months after valve implantation. Pacemaker (PM)-and Implantable Cardioverter Defibrillator (ICD)-related IE were considered healthcare-associated if they occurred within 1-year of device insertion [1,5,6]

Minor clinical criteria included fever >38.0°C, predisposition to IE, and vascular or immunologic phenomena. Major criteria included typical microorganisms isolated from two separate blood cultures and evidence of endocardial involvement on echocardiography [1,5] The diagnosis of definite IE requires two major criteria, or one major and three minor criteria. St. Thomas’ minor criteria were also employed to improve diagnostic yield [8]

IE was classified as acute, if it evolved with sudden onset and rapid progression, presenting within 1-month; subacute, with symptoms evolving between 1- and 6-months after onset; and chronic, with symptoms persisting for more than 6-months [1,5,6]

Embolic events were diagnosed clinically and/or radiologically. All patients with definite IE had systematic imaging techniques performed at the study site, usually contrast-enhanced computed tomography scans, for evaluation of emboli to the central nervous system, abdomen (in left-sided endocarditis), and thorax (in device-related and right-sided endocarditis) [1,5,6]

Comorbidities were diagnosed based on medical records. Acute kidney injury was defined as a creatinine clearance rate <60 mL/min/1.73 m^2^ if not previously present[1,5,6]

Surgical indications were based on the ESC 2005 guidelines (and its subsequent versions) for the management of IE, including heart failure, uncontrolled infection, vegetation >10 mm, or evidence of embolic events [1]

Prior antibiotic use referred to the administration of antibiotics at any time before IE diagnosis [1,5,6]

### Echocardiographic data

Transthoracic Echocardiography (TTE) was routinely performed in all patients. Transesophageal Echocardiography (TEE) was performed to detect cases with negative TTE results, to complement TTE data, and in those with prosthetic or device-related IE. Both TTE and TEE were performed on site. The variables included in the standardized case report form were the presence of vegetation, regurgitation, abscess, leaflet perforation, pseudoaneurysm, fistulae, left ventricular ejection fraction and systolic pulmonary artery pressure.

### Microbiological data

Peripheral venous blood cultures were collected using sterile techniques, with a minimum of two sets, as per institutional protocol, and incubated for 5-days in the BACTEC 9240 system (BACTEC/ALERT®, BioMérieux, Durham, NC, USA). Bacterial identification and antimicrobial susceptibility testing were performed using an automated VITEK 2 system (BioMérieux). The minimum inhibitory concentrations of vancomycin and daptomycin were determined using the E-test for methicillin-resistant *Staphylococcus aureus*.

A subset of patients had surgically excised valves studied by 16SrRNA sequencing [9] Serological and molecular analyses of *Coxiella burnetii* and *Bartonella* spp. were performed at an external reference laboratory in cases of culture-negative endocarditis with strong epidemiological suspicion and no reported history of prior antimicrobial use [9]

### Statistical analysis

Qualitative data were expressed as absolute and relative frequencies. Quantitative data were presented as frequencies, mean ± standard deviation, median, and interquartile range. Statistical analyses were conducted using Jamovi®, version 1.2.2. Statistical significance was set at p<0.05. Categorical data were described as frequencies and percentages. The normality of the numerical variables was assessed using the Kolmogorov-Smirnov test. Proportions were compared using the Chi-Squared test, or the Fisher's exact test, where appropriate. The Student's *t*-test was used to compare means.

### Ethical aspects

This study was approved by the institution's Ethics Committee. Informed consent was obtained from each patient or their legal representative, and the study protocol adhered to the ethical guidelines of the Helsinki Declaration of 1975 and its modifications.

## Results

### Number of cases of definitive IE in adults

Between 1 January 2006 and 30 June 2023, our database identified 502 episodes of definite IE in 481 patients (19 patients had two episodes and one patient had three episodes during the study period).

### General features of the cohort

The mean age of the patients in the cohort was 48±17.2 years, with a ratio of approximately two males for each female (327/502, 65.1 % vs. 175/502, 34.9 %). Individuals over 60-years of age accounted for 149/502 (29.7 %) of the sample. A total of 260/501 (51.9 %) patients were referred from other centers.

Native and prosthetic valve IE occurred in 68.5% and 31.5% of cases, respectively. Early prosthetic valve IE occurred in 59/502 ( 11.8%) cases and late prosthetic valve IE in 99/502 ( 19.7%) cases. Community-acquired IE occurred in 324 ( 64.7%) patients, healthcare-associated nosocomial IE in 128 ( 25.5%), and healthcare-associated non-nosocomial IE in 49 (9.8%).

### Patients’ medical history and comorbidities

Half of the patients (50.3 %) had systemic arterial hypertension, 196 (39.0 %) had Congestive Heart Failure (CHF), 107 (21.4 %) had Chronic Kidney Disease (CKD), and 40.6 % had undergone previous cardiac surgery, mostly valve replacement.

The main predispositions for IE were valve prostheses (158/502; 31.5%), RVD in 151/492 ( 30.7%), congenital heart disease in 70/502 ( 13.9%), and previous IE in 63 ( 12.6%) patients (Table 1).

**Table 1:** Comorbidities, past medical history, and predisposition for IE in adult patients with definite IE, 2006‒2023.

Comorbidities, past medical history and predisposition	n/N (%)
Systemic arterial hypertension	237/471 (50.3)
CHF	203/500 (40.6)
HF	196/502 (39)
CKD	107/507 (21.4)
Dyslipidemia	88/448 (19.6)
Atrial fibrillation/flutter	87/493 (17.6)
Diabetes mellitus	76/502 (15.1)
CAD	71/498 (14.3)
Cerebrovascular disease	34/502 (6.8)
Percutaneous coronary intervention	32/499 (6.4)
Coronary artery bypass graft surgery	25/499 (5)
Neoplasia	27/502 (5.4)
COPD	19/499 (3.8)
HIV/AIDS	7/480 (1.4)
Pacemaker	54/502 (10.8)
Implanted cardiac defibrillator	13/502 (2.6)
Others^a^	18 (3.8)
**Predisposition**	
Prosthesis	158/502 (31.5)
RVD	151/492 (30.7)
Congenital heart disease	70/502 (13.9)
Previous IE	63/501 (12.6)
IV drug use	6/502 (1.2)

a Others = collagen disease (3/501 – 0.6 %), cirrhosis (5/501 – 1.0 %), immunosuppressive use (10/450 – 2.2 %) n/N, Absolute number of findings/total number of episodes with available data; IE, Infective Endocarditis; CHF, Congestive Heart Failure; HF, Heart Failure; CKD, Chronic Kidney Disease; CAD, Coronary Artery Disease; COPD, Chronic Obstructive Pulmonary Disease; HIV, Human Immunodeficiency Virus; AIDS, Acquired Immunodeficiency Syndrome; RVD, Rheumatic Valve Disease; IV, Intravenous.

### Clinical and laboratory features

Most endocarditis cases presented acutely (52 %), followed by subacute (41.3 %) and chronic progression (32/496 cases, 6.4 %).

**Fig. 1:**
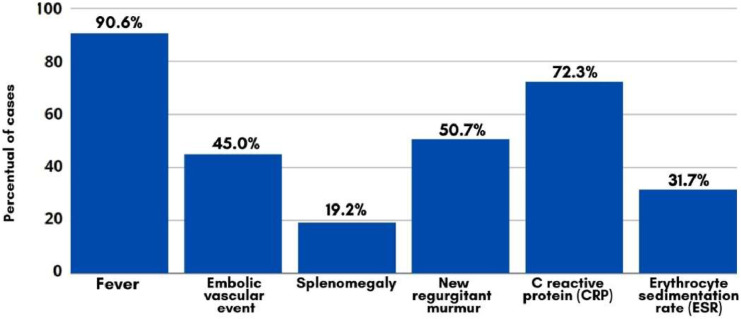
Clinical-laboratory manifestations of IE in adult patients with definite IE, 2006‒2023.

The most common clinical-laboratory manifestation of IE was fever in 454/501(9 0.6%), followed by elevated C-reactive protein (CRP) in 329/455 (7 2.3%) and presence of new regurgitant murmurs (5 0.7%). Approximately one-third of the patients had an elevated erythrocyte sedimentation rate 104/328 (3 1.7%), nearly half had an embolic vascular event (45%), and splenomegaly (1 9.2%) was also a frequent clinical manifestation. Osler's nodes, Janeway lesions, and conjunctival hemorrhage were observed in less tha n 5% of the cases. The clinical manifestations are shown in Fig. 1.

### Microbiological features

**Fig. 2:**
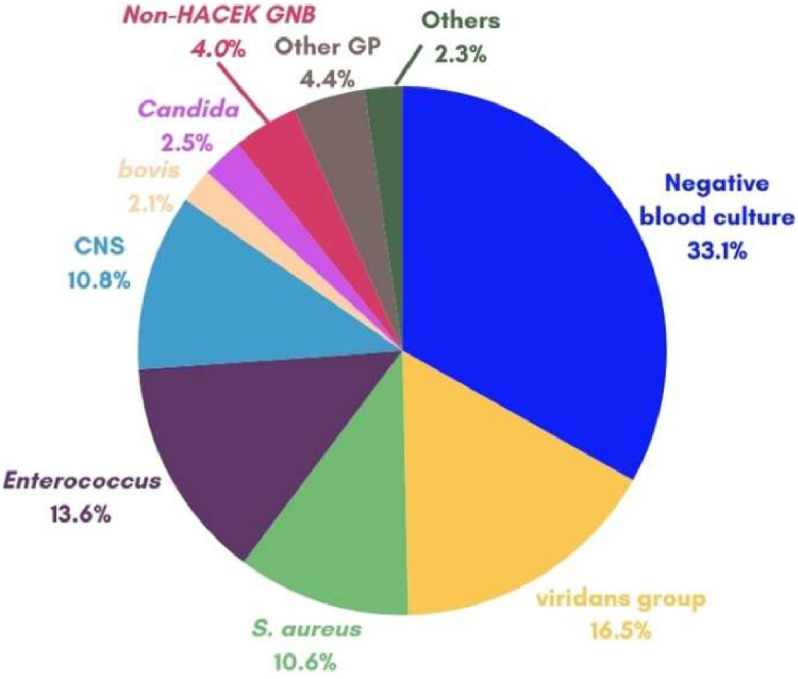
Causative agents in 502 episodes of definite IE in adult patients, 2006‒2023.

Blood cultures were drawn from 495/502 (98.6 %) patients. They were positive in 339/495 (68.5 %) patients. Serology was performed in less than 5 % of the cases (23/502; 4.6 %). Culture-negative IE cases accounted for 33.1 % of all blood cultures collected. Among positive blood cultures, oral streptococci and enterococci were the most frequent, followed by *S. aureus* and coagulase-negative staphylococci. Typical microorganisms such as streptococci of the bovis group and the HACEK group (*Haemophilus species, Aggregatibacter species, Cardiobacterium hominis, Eikenella corrodens and Kingella species)* were infrequent and found in less than 5 % of the cases. Non-HACEK gram-negative bacteria were more frequent than the HACEK group. The etiology of IE is shown in Fig. 2.

Bovis, Bovis group streptococci; viridans group, viridans group streptococci; CNS, Coagulase-Negative Staphylococci; non-HACEK GNB, non-HACEK Gram-Negative bacteria; HACEK, *Haemophilus species, Aggregatibacter species, Cardiobacterium hominis, Eikenella corrodens and Kingella species*; Other GP: *Abiotrophia defectiva* (2); *Corynebacterium diphtheriae* (2); *Corynebacterium amycolatum* (1); *Cutibacterium acnes* (1); *Gemella morbillorum* (3); *Gemella bergeri* (1); *Granulicatella spp* (4); *Kocuria kristinae* (1); *Listeria monocytogenes* (1); *Streptococcus agalactiae* (3); *Streptococcus pneumoniae* (2); *Streptococcus pyogenes* (1). Others: HACEK (5); *Trichosporon s*p (2), *Coxiella burnetii* (2); *Bartonella henselae* (2).

### Echocardiographic features

TTE was performed in 425/500 (85 %) cases, whereas TEE was performed in 390/500 (78 %) cases. Regardless of the involvement of native or prosthetic valves, the most frequent location of vegetations was the Mitral Valve (MV) (221/498, 46.9 %) and the aortic valve (189/499, 37.9 %). PM or ICD infections were reported in 37/501 (7.4 %) cases. The main echocardiographic findings were vegetation (82.9 %), MV vegetations 235/501 (46.9 %), MV regurgitation (221/498; 44.4 %), and aortic valve regurgitation (207/501; 37.9 %). Complications of IE observed on echocardiography included abscesses (15.2 %), perforation of the valve apparatus (17.2 %), and prosthetic valve dehiscence (4.5 %). When comparing early and late prosthetic valve IE and native IE, fistulae were found in 2/59 (3.4 %) of early Prosthetic Valve Endocarditis (PVE), 2/98 (2 %) of late PVE, and 19/345 (5.5 %) of native valve IE patients, respectively, whereas abscesses were found in 11/59 (18.6 %) of early PVE, 24/98 (24.5 %) of late PVE, and 41/345 (11.9 %) of native valve IE patients, respectively. Dehiscence occurred in Early PVE in 5 of 59 (8.5 %) episodes, and in 10 of 95 (10.5 %) episodes of late PVE. The mean pulmonary arterial systolic pressure was 45.9 mmHg. Echocardiographic findings are summarized in Table 2.

**Table 2:** Echocardiographic findings in adult patients with definite IE, 2006‒2023.

Echocardiographic features	n/N (%)
TTE	425/500 (85)
TEE	390/500 (78)
***New moderate to severe regurgitation***	
Aortic	189/499 (37.9)
Mitral	221/498 (44.4)
Tricuspid	82/500 (16.4)
Pulmonary	2/500 (0.4)
**Vegetation**	
Frequency of vegetation (%)	(82.9)
Maximum vegetation size (mm)	(48)
Aortic valve	207/501 (41.3)
Mitral valve	235/501 (46.9)
Tricuspid valve	25/501 (5)
Pulmonary valve	6/501 (1.2)
Chord	10/500 (2)
Catheter	3/501 (0.6)
Device lead	37/50 (7.4)
Myocardial wall	14/501 (2.8)
**Complications**	
Valve perforation	86 (17.2 %)
Abscess	76 (15.2 %)
Cardiac fistula	21/500 (4.2)
Prosthetic dehiscence	19/422 (4.5)
**Quantitative variables**	
EF	M=58.8 % (SD=38)
PASP	M=45.9 mmHg (SD=17.98)

n/N, Absolute number of findings/total number of episodes with available data; TTE, Transthoracic Echocardiogram; TEE, Transesophageal Echocardiogram; EF, Ejection Fraction; PASP, Pulmonary Artery Systolic Pressure; M, Mean; SD, Standard Deviation.

### Complications and outcomes

Among the complications of IE episodes (Table 3), acute heart failure was the most frequent, occurring in more than half of the patients (291/502, 58 %), followed by acute renal failure (156/476, 32.8 %), central neurological events (131/501, 26.1 %), and myocardial or paravalvular abscesses (108/501, 21.6 %). Embolization was also frequently seen, with splenic emboli being the most common with 176/500 cases (35.2 %), followed by emboli to the central nervous system in 131/501 cases (26.1 %). The lungs were affected in 10/35 cases (28.5 %) of device-related endocarditis.

**Table 3:** Complications and outcomes in 502 episodes of definite IE in adult patients, 2006‒2023.

Complications, surgery and outcomes	n/N (%)
Heart failure (HF)	291/502 (58)
Myocardial or paravalvular abscess	108/501 (21.6)
*Prosthetic dehiscence*	20/488 (4.1)
Prosthetic perforation or dysfunction	105/498 (21.5)
Conduction disorder	60/463 (13)
Mycotic aneurysm	49/500 (9.8)
Persistent bacteremia	38/477 (8)
Acute renal failure	156/476 (32.8)
Hemodialysis	51/299 (17.1)
Central neurological event	131/501 (26.1)
Splenic embolization	176/500 (35.2)
Recurrent embolization	28/490 (5.7)
Other embolization^a^	77/502 (15.4)
Pre-operative mechanical ventilation	101/472 (21.4)
Pre-operative inotropic use	122/471 (25.9)
Pre-operative cardiopulmonary arrest	52/479 (10.9)
Intra-aortic balloon use	10/471 (2.1)
Surgical indication	415/502 (82.6)
Cardiac surgery performed	347/415 (83.6)
Overall mortality	125/499 (25)
Mortality in operated patients	74/347 (21.3)
Mortality in non-operated patients	39/68 (57.4)
Mortality in patients who did not have a surgical indication	10/53 (18.9)

a Other embolization = liver (10/498 ‒ 2 %), lungs (27/499 ‒ 5.4 %), peripheral (40/481 – 8.3 %). n/N, Absolute number of findings/total number of episodes with available data.

The overall mortality rate was 125/ 500 (25%) (Table 5). Among all the patients, 415 (82.6 %) had surgical indications. Of these patients, 347 (83.6 %) underwent surgery. Among patients who underwent surgery, the mortality rate was 21.3 % (74/347). Among the 68 patients who had a surgical indication but did not operate, 39/68 died (mortality rate: 57.4 %). This difference was statistically significant (p < 0.001, Chi-Squared test). Only 53 patients did not have surgical indications; among them, 10 died, representing a mortality rate of 18.9 %.

### Comparison of time periods 2006‒2014 and 2025‒2023

Selected variables were compared between two time periods of the cohort study: 2006‒2014, and 2015‒2023. These are presented in Table 4. The number of episodes of IE were 220 and 282 respectively, a 28 % increase in absolute numbers. There were significantly more episodes involving patients older than 60-years, patients on hemodialysis, patients with implantable cardioverter defibrillator, those who had previous cardiac surgery and patients with late prosthetic valve IE. Regarding predisposition, less patients with RVD were seen in the more recent period, and more patients with congenital heart disease. There were more patients with high degree AV block and coagulase negative staphylococcal IE. The frequency of surgical indication, the rate of surgery and the mortality did not change over the years.

**Table 4:** Comparison of selected variables in 502 episodes of infective endocarditis in the time periods 2006‒2014 and 2015‒2023.

Selected Variables	2006‒2014 (n=220)	2015‒2023 (n=282)	Odds ratio	95 % Confidence Interval	p-value
Age(mean±SD)	46.4±16.4	50.0±17.7	**1.012**	**1.002‒1.023**	**0.0229**^a^
Patients >60-years old	54 (24.5 %)	95 (33.7 %)	**1.562**	**1.053‒2.316**	**0.0261**
Male gender	138 (62.7 %)	189 (67.0 %)			0.3165
Early PVE	20 (9.1 %)	39 (13.8 %)			0.1019
Late PVE	29 (13.2 %)	70 (24.8 %)	**2.175**	**1.352‒3.497**	**0.0011**
Previous hemodialysis	16 (7.3 %)	38 (13.5 %)	**1.994**	**1.080–3.681**	**0.0252**
Transfer from another hospital	122 (55.7 %)	138 (48.9 %)			0.1324
Previous heart surgery	75 (34.4 %)	128 (45.4 %)	**1.585**	**1.100–2.282**	**0.0131**
Previous CABG	7 (3.2 %)	18 (6.4 %)			0.1090
Heart failure^b^	81(36.8 %)	115 (40.8 %)			0.3666
Cerebrovascular disease	12 (5.5 %)	22 (7.8 %)			0.2991
Implanted cardiac defibrillator	0	11 (3.9 %)			**0.0032**
Pacemaker	27 (12.3 %)	27 (9.6 %)			0.3330
Diabetes mellitus	26 (11.8 %)	50 (17.7 %)			0.0667
Previous IE	28 (12.7 %)	34 (12.1 %)			0.6626
Congenital heart disease	17/218 (7.8 %)	51 (18.1 %)	**2.610**	**1.461–4.665**	**0.0009**
Rheumatic valve disease	86 (40.6 %)	65 (23.2 %)	**0.443**	**0.300–0.654**	**<.0001**
Community-acquired IE	142 (64.5 %)	182 (64.8 %)			0.9586
Hospital-acquired IE	63 (28.6 %)	65 (23.1 %)			0.1609
Healthcare-related non-hospital acquired IE	6.9 %	12. 1%	1.863	0.987–3.516	0.0520
Valve perforation	40 (18.3 %)	46 (16.4 %)			0.5775
Fistulae	8 (3.7 %)	13 (4.6 %)			0.6032
Perivalvular abscess	28 (12.8 %)	48 (17.0 %)			0.1899
Central nervous system event	60 (27.3 %)	71 (25.3 %)			0.6121
High degree AV block	16 (8.8 %)	44 (15.6 %)	**1.907**	**1.040–3.494**	**0.0345**
Acute renal failure	56 (28.9 %)	100 (35.5 %)			0.1320
*S.aureus* etiology	22 (10.0 %)	30 (10.6 %)			0.8159
CNS	15 (6.8 %)	38 (13.5 %)	**2.128**	**1.138–3.980**	**0.0160**
Enterococcal etiology	23 (10.5 %)	42 (14.9 %)			0.1416
BCNE	76 (34.5 %)	100 (35.5 %)			0.8311
Surgical indication	191 (86.8 %)	229 (81.2 %)			0.0915
Surgery performed	158 (71.8 %)	195 (69.1 %)			0.5160
In-hospital death	57 (25.9 %)	68 (24.2 %)			0.6607

SD, Standard Deviation; PVE, Prosthetic Valve Endocarditis; CABG, Coronary Artery Bypass Grafting; IE, Infective Endocarditis; AV, Atrioventricular; BCNE, Blood Culture Negative Endocarditis; CNS, Coagulase Negative Staphylococci.

a Student's *t*-test.

b Heart failure before the present episode of infective endocarditis.

## Discussion

IE is a severe condition with a high risk of complications and mortality, requiring specialized multidisciplinary management. This study presented a series of 502 episodes of definite IE in adult patients, at a federal public hospital in Rio de Janeiro, Brazil. A comparison between two time periods (2006‒2014 and 2015‒2023) was also made, showing interesting features, such as less RVD, and more episodes of late PVE and IE affecting patients with congenital cardiac disease.

Few case series of IE have been reported from Brazilian centers [10, 11, 12] and from other low- and middle-income countries. To facilitate the discussion, we compiled Table 1, Table 2, Table 3, Table 4 from the literature (Supplementary Tables).

Our study revealed a mean patient age of 48-years, consistent with the literature indicating that younger age groups are more commonly affected in the low- and middle-income countries [13, 14, 15, 16, 17] This finding is associated with the frequent presence of RVD as a predisposing factor, leading to earlier onset of IE [15,16] Rheumatic valve disease was also prevalent in other Brazilian studies [10,11] In contrast, a larger IE series by Murdoch et al., [2] which included 2781 adults from 58 hospitals across 25 countries from 2000 to 2005, reported a median age of 57.9-years. The EURO-ENDO study, [18] including 3116 patients from 2016 to 2018, predominantly from European centers, reported a median age of 65-years. Recent case series from European countries have also reported higher mean ages compared to our study [19,20] The most common underlying cardiac conditions in this study's cohort were rheumatic valve disease (30.7 %) and congenital heart disease (13.9 %) similar to the French Polynesia, [21] Saudi Arabia, [16] and China [15] (Supplementary Table 1).

IE on native valves occurred in 68.5 % of patients, with a higher incidence on the aortic and mitral valves, similar to observations in both developed and developing countries [2,4,10,12,15, 16,18, 19,22] Prosthetic valve endocarditis accounted for 31.47 % of cases, like findings in Italy, [20] Saudi Arabia, [16] Turkey, [23] and other Brazilian studies (27.1 %; 48.4 %) [10,11] Despite the predominant involvement of native valves, the incidence of prosthetic valve endocarditis has increased, consistent with the rising number of valve surgeries in recent years [24] In low- and middle-income countries, the incidence of prosthetic valve endocarditis is significantly lower than that of native valve endocarditis, possibly due to limited access to healthcare and fewer valve replacements [15,23] Over the years, a significant increase in late PVE and implanted cardiac defibrillators was seen in our study, as well as in congenital heart disease, whilst there was a decrease in RVD. The incidence of IE in implantable cardiac electronic devices has shown an increasing trend owing to higher device insertion rates, population aging, and increased comorbidities. In this study, it represented approximately 8 % of all cases over the past 17-years, a proportion like that reported in Argentina, China, and Turkey [13,14,24] (Supplementary Table 2).

The most relevant classical signs and symptoms observed were fever (90.6%) and heart murmur (50.7%), consistent with other studies (Supplementary Table 4). Classic Oslerian manifestations of endocarditis were present in only 3 % of patients upon hospital admission, corroborating findings from other Brazilian [11] and Chinese [15] studies. Although embolic manifestations were frequent (45 %) in this cohort, other studies reported a lower proportion despite a significant number of cases; [11, 12,15,19,25, 26] we highlight that, in our study, emboli were often detected radiologically rather than clinically. This underscores the recommendation for echocardiography in cases of bacteremia and screening for embolic events, even in asymptomatic patients [27] As shown in Supplementary Table 4, embolization was the most frequently reported complication, although not all studies specified the affected areas.

The proportion of patients who underwent TEE in this cohort (78%) was significantly higher than that reported in a Chinese study ( 12.8%). This contrasts with the 2015 ESC guidelines on IE, [1] which recommend performing TEE to exclude perivalvular complications, even if TTE shows findings compatible with IE. The high frequency of TEE in our study reflects the setting of a highly specialized surgical cardiology hospital. The lower utilization of TEE in other studies may have led to lower detection rates of perivalvular complications such as valve perforation and perivalvular abscess, potentially resulting in missing these diagnoses in patients with subtle or early valvular lesions.

The number of positive blood cultures in this study was lower than that in studies from developed countries [19,23,25,28] but higher than those from developing countries, [14, 15, 16] as demonstrated in Supplementary Table 3. Brazilian studies reported blood culture positivity rates of 66.5 %, [10] 76.9 %, [13] and 76.6 % [12] The low rate of microbiological detection may be related to antibiotic use before blood culture collection, as previously described by our group, where the rate of prior antibiotic use reached 75 % [9] Additionally, over half of the patients in our center were referred from other medical institutions, where antibiotic treatment is often initiated before blood cultures are collected. Oral streptococci were the primary etiological agents isolated in this study. Likewise, studies from Argentina and China [13,14] show the predominance of oral streptococci, in contrast to those in developed countries, [22,28], where *S. aureus* has become the main aetiology (Supplementary Table 3). The increase in staphylococcal infections is primarily attributed to the high incidence of infections among intravenous drug users, [4,22] patients undergoing hemodialysis, [22] and older patients with comorbidities. Noteworthily, the proportion of intravenous drug users and octogenarians was low in our cohort compared to developed countries [22,28,29] Over the years, our study did not show a change in the frequency of *S.aureus* IE, nor of BCNE, but coagulase negative staphylococci increased, possibly associated with healthcare acquisition in late PVE.

Surgery is indicated to prevent progressive and irreversible structural damage and is justified in patients at high risk where cure with antimicrobial therapy alone is unlikely and in those who do not have comorbidities or severe complications that make recovery prospects remote [1,6] This review revealed that a wide range of patients underwent surgery for IE, from 17.2 % in Japan [30] to 69.7 % in Turkey [23] The study with the lowest surgical indication rate in this review was Japan [30] which despite also being a high-income country, has an older average population age (69.1±14-years) with multiple comorbidities, often reducing the drive for surgical intervention.

Unfortunately, our cohort did not have sufficient power to compare patients who underwent early intervention with those who underwent late surgery. However, our study provided evidence supporting the assumption that surgically treated patients have better outcomes than conservatively managed patients. Valve replacement or valve repair surgery is recommended (and reduces mortality) in cases of IE with complications such as embolic events, CHF, and valvular abscesses [1,3,6,28]

Despite improvements in the diagnostic accuracy, medical therapy, and surgical techniques, IE mortality rates remain relatively high worldwide. In our study, we observed an overall hospital mortality rate of 25 %, which is similar to the rates observed in Japan [30] (26.1 %) and Turkey [25] (22.6 %). However, these results were lower than those of other studies, such as French Polynesia [21] (37 %) and Spain [19] (34.7 %). This may be due to the younger age of our patients and the lower proportion of infections caused by *S. aureus*. Oral streptococcal species were the most frequently identified microorganisms in our study, which may have contributed to the favorable outcomes. Available studies in Brazil have reported higher mortality rates than ours (Minas Gerais, 32 %; São Paulo, 33 %; and Rio Grande do Sul, 41.9%) [10, 11, 12] These higher rates may be attributed to differences in patient profiles, with a prevalence of multiple comorbidities, and differences in hospital profiles, as ours is a referral center in cardiology and cardiac surgery[10, 11, 12,21, 22]

The main limitation of our study was its retrospective, single-center nature at a cardiac surgery referral centre, which may not represent the profile of the entire Brazilian and South American healthcare systems. Therefore, our findings cannot be generalized, and the results may have been influenced by this limitation. A significant strength of our study is that it included one of the largest cohorts of adult patients with definite IE, with prospectively collected data, in Latin America.

## Conclusion

This study presents the IE profile and mortality analysis in a large cohort of patients spanning a 17-year period, representing a rare initiative in a middle-income country. Blood culture-negative and oral streptococcal endocarditis were frequent and affected younger patients. Over the years, coagulase negative staphylococci became more frequent as causative agents, and older patients with more complex background occurred: late prosthetic valve IE, patients who had ICD, who were on hemodialysis, and who had been submitted previously to cardiac surgery.

The high mortality rate observed in this cohort underscores the importance of studies on IE, as they provide a better understanding of clinical and microbiological characteristics, as well as factors associated with unfavorable prognosis. This could contribute to the development of improved strategies in the management of IE.

## Ethical approval statement

The study was approved by the Institutional Review Board under n° 65,137,922.7.0000.5272 on 22^nd^ November 2022.

## Funding

We thank Fundação Pró Coração (FUNDACOR) for grants supporting the Endocarditis project and individual grants to the authors MGBC, TVPAA and NAPF; we thank Fundação Carlos Chagas Filho de Amparo à Pesquisa do Estado do Rio de Janeiro (FAPERJ) for a grant to NAPF, and Mestrado Profissional em Ciências Cardiovasculares, Instituto Nacional de Cardiologia, Ministerio da Saude do Brasil.

## Conflicts of interest

The authors declare no conflicts of interest.

## Appendix Supplementary materials

Image, application 1
