# Supplementary material for: Contemporary cohort study in adult patients with infective endocarditis
Source: Braz J Infect Dis. 2025 Apr 2;29(3):104521. doi: 10.1016/j.bjid.2025.104521 (PMC11999418; doi:10.1016/j.bjid.2025.104521)
Supplement: Supplementary file 1 [file mmc1.docx]

**BJID-D-24-00245_Supplementary Material**

**Supplementary Material - Review of IE studies in low-, middle- and high-income countries**

**Table 1 G**eneral features, patient demographics, comorbidities and predisposition in series of infective endocarditis.

| **Year** | **GENERAL FEATURES** | | | **PATIENT DEMOGRAPHICS** | | | **COMORBIDITIES (%)** | | | | **PREDISPOSITION (%)** | | | |
| --- | --- | --- | --- | --- | --- | --- | --- | --- | --- | --- | --- | --- | --- | --- |
|  | **Author/Country/**  **Reference** | **Type**  **Of Hospital** | **Follow-up (years)** | **Number of patients** | **Age**  **(average; ±SD)** | **Sex**  **(male %)** | **Cardiac failure** | **Diabetes mellitus** | **Chronic renal**  **failure** | **HD** | **Rheumatic vavlopathy** | **Congenic Heart Disease** | **Intravenous drug use** | **Previous IE** |
| **2014** | Pallotto, C., et al.  Perugia, Italy[22] | University | 39 | 232 | 62.1±17.6 | 159 (68.5) | - | 43/232 (18.5) | - | 9/232 (3.9) | 8/232 (3.5) | 6/232 (2.6) | 12/232 (5.2) | 10/232 (4.3) |
| **2014** | *Siciliano,* et al*.,*  São Paulo, Brazil[12] | Tertiary | 5 | 221 | 53 ± 18.4 | 144 (65) | 78 (35.3) | 25 (11.3) | 15 (6.8) | - | 29 (13.1) | 22 (9.9) | - | 9 (4) |
| **2015** | Hase, R., et al.  Kamogawa, Japan[24] | Tertiary | 14 | 180 | 69.1 ± 14 | 109 (60.6) | 50 (27.8) | 25 (13.8) | - | 19 (10.6) | - | - | - | - |
| **2017** | Zhu, W; Zhang, J.  Taiwan, China[16] | University | 8.5 | 196 | 43.5 (29.0–55.8) | 136 (69.4) | - | - | - | - | 19 (9.7) | 72 (36.7) | - | - |
| **2018** | Kashef, M A, et al.  New England, USA[32] | University | 4 | 170 | 60.0 ± 17.9 | 113 (66.5) | 38 (22.4) | 38 (22.4) | 61 (35.9) | 27 (15.9) | - | - | 23 (13.5) | 14 (8.2) |
| **2018** | Nunes, et al.,  Minas Gerais, Brazil[13] | Tertiary | 11.6 | 203 | 48.2± 16.6 | 125 (62) | - | 25 (12.3) | 28 (13.7) | 28 (13.7) | 78 (38.4) | - | - | 20 (10) |
| **2019** | Agus, H Z, et al.  Istanbul, Turkey[26] | Tertiary | 10 | 155 | 58 (43-66) | 103 (66.5) | 19 (12.3) | 22 (14.2) | 23 (14.8) | 3 (1.9) | 15 (9.7) | 4 (2.6) | 5 (3.2) | - |
| **2020** | Blanchard, et al.,  Taitii, French Polynesian[23] | Tertiary | 3 | 190 | 55 ± 17 | 68 (65) | - | - | 19 (18) | - | 35(33) | - | - | 5 (5) |
| **2020** | Holland, D.J., et al., Australia[27] | Tertiary | 5.2 | 204 | 57.4 ± 18 | 138 (67.6) | 32 (15.7) | 57 (27.9) | 44 (21.6) | 15 (7.4) | 11 (5.4) | 5 (2.5) | 47 (24.5) | 16 (7.8) |
| **2020** | Kim, J. H., et al.,  Seul, South Korea[29] | University | 11.7 | 419 | 56 (43–68) | 275 (65.6) | 29 (6.9) | 77 (18.4) | 43 (10.3) | 23 (5.5) | - | - | - | 21 (5.0) |
| **2020** | Tagliari*,* et al.,  Porto Alegre, Brazil[14] | Tertiary | 5 | 167 | 60 | 110 (66) | 57 (34.1) | 48 (29) | 35 (21) | - | - | - | - | - |
| **2020** | Wu, Z., et al.,  Hangzhou, China[17] | University | 10 | 409 | 48 ± 16 | 262 (64.4) | 221 (54.3) | 37 (9.1) | 60 (16.5) | 14 (3.4) | 69 (17) | 105 (25.8) | 3 (0.7) | - |
| **2021** | Acibuca, A., et al.  Adana, Turkey[30] | General | 10.7 | 139 | 55 ± 16 | 83 (59.7) | - | 46 (33.1) | 45 (32.4) | - | 5 (3.6) | - | 1 (0.7) | 1 (0.7) |
| **2021** | Pecoraro, et al.  Cape Town, South Africa[19] | Tertiary | 1.2 | 140 | 39.6 (12.8) | 94 (67.1) | - | 7 (5) | - | - | - | - | 10 (7.1) | - |
| **2021** | Ballesta, AIP, et al.,  Spain[21] | University | 17 | 101 | 64.5 ± 16 | 69 (68.3) | 30 (29.7) | 30 (29.7) | 15 (14.8) | - | - | - | - | - |
| **2021** | Barry, M, et al.  Riad, Saudi Arabia[18] | Public | 4 | 345 | 48.09 ± 18.94 | 228 (67.1) | - | - | - | 12 (3.5) | 51 (15) | 32 (9.4) | - | - |
| **2021** | Kazelian, L.R. et al.  Buenos Aires, Argentina[15] | General | 30 | 252 | 46.4± 16 | 173 (68.7) | - | - | - | - | 31 (12.3) | 88 (34.9) | 7 (2.8) | 36 (14.3) |
| **2022** | Liaqat, F., et al.  Bronx, NY, USA[25] | Tertiary | 10.7 | 182 | 54 (44–67) | 112 (61.5) | 33 (18.1) | 59 (32.4) | 17 (9.3) | 26 (14.3) | 5 (2.8) | 13 (7.1) | 46 (25.3) | 19 (10.4) |

IE, Infective endocarditis; SD, standard deviation; HD, haemodialysis.

**Table 2** Echocardiographic features in series of infective endocarditis.

| **Year** | **Author/Country/Reference** | **ECOCARDIOGRAPHIC CHARACTERISTICS (%)** | | | **LOCATION OF IE (%)** | | | | | | |
| --- | --- | --- | --- | --- | --- | --- | --- | --- | --- | --- | --- |
|  |  | TTE | **TEE** | **Presence of vegetation** | **Native**  **valve** | **Aortic** | **Mitral** | **Tricuspid** | **Pulmonary** | **Prosthesis** | **Cardiac devices** |
| **2014** | Pallotto, C., et al.  Perugia, Italy[22] | 180 (77.5) | 157 (67.7) | - | - | 96/217 (44.2) | 91/217 (41.9) | 12/217 (5.5) | - | 75/232 (32.3) | 3/232 (1.3) |
| **2014** | Siciliano, et al.,  São Paulo, Brazil[12] | - | - | 171/219 (78) | 114 (51.6) | 209 (94.5) | 209 (94.5) | 12 (5.4) | 12 (5.4) | 107 (48.4) | - |
| **2015** | Hase, R., et al.  Kamogawa, Japan[24] | 180 (100) | 132 (73.3) | 128 (71.1) | - | 58 (32.2) | 76 (42.2) | 1 (0.6) | 1 (0.6) | 35 (19.4) | - |
| **2017** | Zhu, W; Zhang, J.  Taiwan, China[16] | - | - | - | - | - | - | - | - | - | 8 (4,1) |
| **2018** | Kashef, M A, et al.  New England, USA[32] | 125 (73.5) | 100 (58.8) | 143 (84.1) | 127 (74.7) | 41 (28.7) | 59 (41.3) | 8 (5.6) | 1 (0.7) | 43 (24.3) | - |
| **2018** | Nunes, et al.,  Minas Gerais, Brazil[13] | 46 (22.8) | 157 (77.2) | 180 (88.2) | L: 113 (55.7) | 43 (21.2) | 70 (34.5) | - | - | 55 (27.1) | R /PM: 44 (21.6) |
| **2019** | Agus, H Z, et al.  Istanbul, Turkey[26] | 155 (100) | 145 (93.5) | TTE: 145 (93,5)/ TEE: 103 (66,5); | 93 (60) | 67 (43.2) | 84 (54.2) | 6 (3.9) | 1 (0.6) | 55 (35.5) | 7 (4.5) |
| **2020** | Blanchard, et al.,  Taitii, French Polynesian[23] | - | 98 (93) | - | - | 54 (51) | 56 (53) | 3 (3) | 4 (4) | - | - |
| **2020** | Holland, D.J., et al.*,* Australia[27] | 196 (96.1) | 144 (70.6) | - | 156 (76.4) | 76 (37.3) | 92 (45.1) | 27 (13.2) | 3 (1.5) | 31 (15.2) | 6 (2.9) |
| **2020** | Kim, J. H., et al.  Seul, South Korea[29] | - | - | - | - | 181 (43.2) | 257 (61.3) | 34 (8.1) | 16 (3.8) | 63 (15) | - |
| **2020** | Tagliari, et al.,  Porto Alegre, Brazil[14] | - | - | 151 (90.4) | 122 (73) | 91 (54.5) | 73 (43.7) | 12 (8.7) | - | - | 8 (4.8) |
| **2020** | Wu, Z., et al.,  Hangzhou, China[17] | - | 52 (12.8) | 350 (86) | 370 (90.9) | 143 (35.1) | 134 (32.9) | - | - | 37 (9.1)+ | 37 (9.1)+ |
| **2021** | Acibuca, A., et al.  Adana, Turkey[30] | - | - | TTE: 90 (64,7/ TEE: 138 (99,3) | - | 36 (25.9) | 75 (54) | 11 (7.9) | 1 (0.7) | - | 5 (3.6) |
| **2021** | Pecoraro, et al.  Cape Town, South Africa[19] | - | - | - | - | - | - | - | - | - | - |
| **2021** | Ballesta, AIP, et al.  Spain[21] | 100 (99) | - | - | 83 (82) | 41 (40.5) | 47 (46.5) | 8 (7.9) | 3 (3) | 15 (14) | 2 (2) |
| **2021** | Barry, M, et al.  Riad, Saudi Arabia[18] | 340 (100) | 224 (65.8) | - | 111 (32.6) | 79 (23) | 122 (36) | 17 (5) | 14 (4) | 109 (32) | - |
| **2021** | Kazelian, L.R. et al.  Buenos Aires, Argentina[15] | 246 (97.6) | 79 (31) | 204 (80.9) | - | 94 (46) | 48 (23.5) | 36 (17.4) | 6 (2.9) | 27 (10.7) | 16 (6.3) |
| **2022** | Liaqat, F., et al.  Bronx, NY, USA[25] | 81 (44.4) | 102 (55.6) | (79.1) | 164 (90.1) | (19.8) | (32.4) | (18.1) | (2.2) | 15 (8.2) | - |

IE, Infective endocarditis; TTE, Transthoracic Echocardiography; TEE, Transesophageal Echocardiography; L, left; R, Right; PM, Pacemaker; +IE Prosthesis or cardiac devices.

**Table 3** Microbiological features in series of infective endocarditis.

| **Year** | **Author/Country/Reference** | **MICROBIOLOGICAL CHARACTERISTICS (%)** | | | | | | | | | | | |
| --- | --- | --- | --- | --- | --- | --- | --- | --- | --- | --- | --- | --- | --- |
|  |  | **Blood culture +** | **Blood culture -** | **Staphylococcus spp.** | ***S. aureus*** | **CNS** | **Streptococcus spp** | **Oral streptococci/ viridans** | **Bovis group streptococci (*S. gallolyticus*)** | **GBS** | **Enterococci** | **Fungus** | **HACEK** |
| **2014** | Pallotto, C., et al.  Perugia, Italy[22] | - | 34 (14.7) | - | 55 (23.7) | 24 (10,3) | - | 35 (15.1) | 18 (7.8) | - | 24 (10.3) | 6 (2.6) | - |
| **2014** | Siciliano*,* et al.*,*  São Paulo, Brazil[12] | 170 (77) | 51 (23.1) | - | 14 (8.2) | 6 (3.5) | 11 (6.5) | 81 (47.6) | 17 (10) | - | 20 (11.7) | 1 (0.4) | 13 (7.7) |
| **2015** | Hase, R., et al.  Kamogawa, Japan[24] | - | 9 (5) | - | (49 (27.2) | 21 (11.7) | - | 40 (22.2) | - | 11 (6.1) | 11 (6.1) | - | - |
| **2017** | Zhu, W; Zhang, J.  Taiwan, China[16] | 96 (49) | - | - | 18 (18.7) | - | 51 (53.1) | 45/51 (42.7) | - | - | 10 (10.4) | 2 (2) | - |
| **2018** | Kashef, M A, et al.  New England, USA[32] | - | 10 (5.9) | - | 68 (40) | 10 (5.9) | - | 29 (17.1) | 2 (1) | 10 (5.9) | 26 (15.3) | - | 1 (0.5) |
| **2018** | Nunes, et al.,  Minas Gerais, Brazil[13] | 135 (66.5) | 68 (33.5) | 69 (51.1) | 37 (27.4) | - | 30 (21.5) | - | - | - | 15 (11.1) | * | * |
| **2019** | Agus, H Z, et al.  Istanbul, Turkey[26] | - | 73 (48.4) | 52 (33.5) | 15 (9.6) | 37 (23.8) | 10 (6.5) | - | - | - | 8 (5.2) | 3 (1.9) | - |
| **2020** | Blanchard, et al.,  Taitii, French Polynesian[23] | - | 14 (14) | 40 (38) | 37 (35) | 3 (3) | 32 (30) | 6 (6) | 4 (4) | 3 (3) | 6 (6) | 0 (0) | 1 (1) |
| **2020** | Holland, D.J., et al.*,* Australia[27] | 181 (88.7) | 23 (11.3) | - | 92 (45.1) | 18 (9.3) | - | 24 (11.8) | - | - | 18 (9.3) | 0 (0) | 2 (2.1) |
| **2020** | Kim, J. H., et al.  Seul, South Korea[29] | 309 (73.7) | 110 (26.3) | - | 66 (15.8) | 32 (7.6) | 147 (35.1) | - | - | - | 36 (8.6) | 4 (1) | 2 (0.5) |
| **2020** | *Tagliari,* et al.,  Porto Alegre, Brazil[14] | 128 (76.6) | 39 (23.3) | 14 (8.3) # | 32 (19.2) | 17 (10.2) | 22 (13.2) | 16 (9.6) | 14 (8.3) # | 14 (8.3) # | 20 (12) | 7 (4.2) | 14 (8.3) # |
| **2020** | Wu, Z., et al.,  Hangzhou, China[17] | 222 (54.5) | 185 (45.5) | 80 (19.7) | 40 (9.8) | 28 (6.9) | 100 (24.6) | - | - | - | 5 (1.2) | - | - |
| **2021** | Acibuca, A., et al.  Adana, Turkey[30] | - | 30 (21.6) | - | 22 (15.8) | 42 (30.2) | 15 (10.8) | 4 (2.9) | 1 (0.7) | - | 9 (6.5) | 3 (1.9) | 1 (0.7) |
| **2021** | Pecoraro, et al.  Cape Town, South Africa[19] | - | 75 (53.6) | - | 64 (46) | 4 (3) | - | 38 (27) | - | - | - | - | - |
| **2021** | Ballesta, AIP, et al.  Spain[21] | 95 (94) | 5 (4,9) | - | 35 (34) | 12 (11) | 27 (26.7) | - | - | - | 11 (10.8) | 1 (0,9) | - |
| **2021** | Barry, M, et al.  Riad, Saudi Arabia[18] | 177 (52.1) | 163 (47.9) | 80/144 (55.5) | 53/80 (66.2) | 27/80 (33.7) | 37/144 (25) | 29/37 (78.4) | 1/37 (2.7) | - | 27/144 (18.8) | 5/177 (2.8) | - |
| **2021** | Kazelian, L.R. et al.  Buenos Aires, Argentina[15] | 186 (73.8) | - | - | 45 (17.8) | - | - | 57 (22.6) | 8 (3.1) | - | 14 (5.5) | 5 (2.7) | - |
| **2022** | Liaqat, F., et al.  Bronx, NY, USA[25] | 153 (84.1) | 29 (16) | 87/139 (62.6) | 69/87 (79.3) | 18/87 (20.7) | 37/139 (26.6) | 16/37 (43.2) | 3/37 (8.1) | 5/37 (16.5) | 12/139 (8.6) | 4 (2.6) | - |

+, positive; -, negative; HACEK, *Haemophilus spp., Aggregatibacter spp., Cardiobacterium hominis, Eikenella corrodens, Kingella species*; CNS, coagulase-negative staphylococci; GBS, Group B Streptococci (*S*. *agalactiae*).

* HACEK, Fungus and Other Gram positives were grouped together.

# Others (*Staphylococcu*s spp, bovis group streptococci, GBS and HACEK).

**Table 4** Clinical-laboratory features, complications and outcomes in infective endocarditis.

| **Year** | **Author/Country/Reference** | **CLINICAL SYMPTOMS (%)** | | | | | **COMPLICATION (%)** | | | | | | **OUTCOMES** | | |
| --- | --- | --- | --- | --- | --- | --- | --- | --- | --- | --- | --- | --- | --- | --- | --- |
|  |  | **Fever** | **Presence of new murmur** | **Embolic events** | **High ESR** | **High CRP** | **Abscess** | **Perforation** | **Sepsis/Septic Shock** | **Embolism** | **Congestive Heart Failure** | **Acute Renal Failure** | **Surgical Indication** | **Surgery** | **Mortality** |
| **2014** | Pallotto, C., et al.  Perugia, Italy[22] | - | - | - | - | - | 34 (14.6) | 14 (6) | 7 (3) | 107 (46.1) | - | - | - | 68/232 (29.3) | 41/217 (18.9) |
| **2014** | *Siciliano,* et al*.,*  São Paulo, Brazil[12] | 197 (89) | - | - | - | 92 (41.6) | 59 (26.7) | - | 80 (36.2) | 76 (34.4) | - | 114 (51.5) | - | 116 (52.4) | 72 (33) |
| **2015** | Hase, R., et al.  Kamogawa, Japan[24] | - | - | - | - | - | - | - | - | - | - | - | - | 31 (17.2) | 47 (26.1) |
| **2017** | Zhu, W; Zhang, J.  Taiwan, China[16] | 168 (85.7) | 119 (60.7) | - | 89 (64.5) | 109 (70.8) | - | - | - | - | 40 (20.4) | 24 (12.2) | - | - | - |
| **2018** | Kashef, M A, et al.  New England, USA[32] | - | - | - | - | - | 16 (9.4) | 19 (11.2) | - | 42 (27.7) | - | - | - | 47 (27.6) | 25 (14.7) |
| **2018** | Nunes, et al.,  Minas Gerais, Brazil[13] | 162 (80) | 152 (75) | 29 (14.2) | - | 174 (85) | 13 (6.4) | 35 (17.3) | - | 22 (10.8) | 79 (39) | - | - | 111 (55) | 65 (32) |
| **2019** | Agus, H Z, et al.  Istanbul, Turkey[26] | 102 (65.8) | - | 1 (0.6) | - | 78 (50.3) | 19 (12.3) | 25 (16.1) | 6 (3.9) | 4 (2.6) | - | 18 (11.6) | 108 (69.7) | 108 (69.7) | 35 (22.6) |
| **2020** | Blanchard, et al.,  Taitii, French Polynesian[23] | - | - | - | - | - | 7 (5) | - | P: 19 (18)  C: 29 (27) | 49 (47) | 25 (23) | - | 73 (69) | 38 (52) | 39 (37) |
| **2020** | Holland, D.J., et al.*,* Australia[27] | - | - | - | - | - | 23 (11.3) | - | - | 110 (53.9) | 80 (39.2) | 126 (67.5) | - | 81 (39.7) | - |
| **2020** | Kim, J. H., et al.  Seul, South Korea[29] | 310 (74) | - | 35 (8.4) | 61 (14.5) | 46 (11) | - | - | 294 (70.2) | 35 (8.4) | 58 (13.8) | 62 (14.8) | 273 (83.4) | - | 61 (14.6) |
| **2020** | Tagliari, et al*.*,  Porto Alegre, Brazil[14] | 141 (84.3) | 66 (39.5) | C: 30 (18)  P: 35 (21) |  |  | 16 (9.6) | - | - | - | - | - | 82 (49.1) | 81 (49) | 70 (41.9) |
| **2020** | Wu, Z., et al.,  Hangzhou, China[17] | 365 (89.7) | - | 74 (18.2) | - | - | 30 (7.4) | 72 (17.8) | - | 125 (30.7) | - | - | 403 (99) | 235 (57.7) | 43 (10.6) |
| **2021** | Acibuca, A., et al.  Adana, Turkey[30] | 77 (55.4) | - | 10 (7.2) | - | - | - | - | - | 55 (39.6) | 31 (22.3) | 11 (7.9) | - | 91 (65.6) | 42 (30.2) |
| **2021** | Pecoraro, et al.  Cape Town, South Africa[19] | - | - | - | - | - | - | - | - | - | - |  | - | 39/65 (60) | 25/140 (17,8) |
| **2021** | Ballesta, AIP, et al.  Spain[21] | 101 (100) | (64) | 29 (28.9) | - | - | - | - | 47/101 (46.5) | Brain: (16.7)  Spleen: (7) | 76/101 (75.2) | - | 25 (34.6) | 25 (24.8) | 35 (34.7) |
| **2021** | Barry, M, et al.  Riad, Saudi Arabia[18] | 320 (94) | 61 (18) | - | - | - | - | - | - | 18 (5) | 30 (9) | 67 (20) | - | 89 (26.2) | 23 (6.8) |
| **2021** | Kazelian, L.R. et al.  Buenos Aires, Argentina[15] | 203 (80.6) | - | - | - | - | 18 (12.5) | - | 40 (27.8) | 55 (38.2) | 93 (64.6) | 59 (41) | 158 (64.5) | 89 (35.3) | 77 (30.6) |
| **2022** | Liaqat, F., et al.  Bronx, NY, USA[25] | 105 (58) | 39 (21.4) | - | - | - | - | - | - | Lungs: 41 (22.5)  CNS: 27 (14.8)  Others: 23 (12.6) | - | 51 (28) | - | 40 (22) | 33 (18.1) |

ESR, erythrocyte sedimentation rate; CRP, C reactive protein; C, cerebral embolic event; P, peripheral embolic event; CNS, central nervous system.
